# The Pharmacology of Chloral Hydrate

**Published:** 1881-05

**Authors:** 


### THE PHARMACOLOGY OF CHLORAL HYDRATE.

M. Dmitrieff, in a recent inaugural dissertation, published at St. Petersburg, states that he has tested the effect of chloral hydrate, both clinically and experimentally, on unhealthy, badly granulated wounds. By incision of a piece of skin in dogs, and infection of the wound with putrefying matter, he produced unhealthy ulcerated surfaces. Some of these he dressed with one or two per cent. solution of chloral hydrate, while the rest were simply covered with a moist cloth. The first very soon became healthy, and cicatrized before the others. The ulcers, on microscopic examination, were found covered with a layer of micrococci, which disappear after two or three days' dressing with chloral hydrate. These results were confirmed clinically; and the writer has also shown that an equal quantity of a one per cent. solution of cholral hydrate destroyed, in twenty minutes, all mobility of the bacteria in a putrefying infusion of flesh. —*Medical and Surgical Reporter.*

